# Different Lengths of Gestational Exposure to Secondhand Smoke or e-Cigarette Vapor Induce the Development of Placental Disease Symptoms

**DOI:** 10.3390/cells13121009

**Published:** 2024-06-09

**Authors:** Madison N. Kirkham, Christian Cooper, Emily Broberg, Peter Robertson, Derek Clarke, Brett E. Pickett, Benjamin Bikman, Paul R. Reynolds, Juan A. Arroyo

**Affiliations:** 1 Lung and Placenta Laboratory, Department of Cell Biology and Physiology, Brigham Young University, Provo, UT 84602, USAembrob432@byu.edu (E.B.);; 2 Department of Microbiology and Molecular Biology, Brigham Young University, Provo, UT 84602, USA; brett_pickett@byu.edu

**Keywords:** secondhand smoke, e-cigarette, placenta, IUGR, PE

## Abstract

Exposure to cigarette smoke is known to induce disease during pregnancy. Recent evidence showed that exposure to secondhand smoke (SHS) negatively impacts fetal and placental weights, leading to the development of intrauterine growth restriction (IUGR). Electronic cigarettes (eCigs) represent a phenomenon that has recently emerged, and their use is also steadily rising. Even so, the effects of SHS or eCigs during gestation remain limited. In the present study, we wanted to characterize the effects of SHS or eCig exposure at two different important gestational points during mouse pregnancy. C57/Bl6 mice were exposed to SHS or eCigs via a nose-only delivery system for 4 days (from 14.5 to 17.5 gestational days (dGA) or for 6 days (from 12.5 dGA to 17.5 dGA)). At the time of necropsy (18.5 dGA), placental and fetal weights were recorded, maternal blood pressure was determined, and a dipstick test to measure proteinuria was performed. Placental tissues were collected, and inflammatory molecules in the placenta were identified. Treatment with SHS showed the following: (1) a significant decrease in placental and fetal weights following four days of exposure, (2) higher systolic and diastolic blood pressure following six days of exposure, and (3) increased proteinuria after six days of exposure. Treatment with eCigs showed the following: (1) a significant decrease in placental weight and fetal weight following four or six days of exposure, (2) higher systolic and diastolic blood pressure following six days of exposure, and (3) increased proteinuria after six days of exposure. We also observed different inflammatory markers associated with the development of IUGR or PE. We conclude that the detrimental effects of SHS or eCig treatment coincide with the length of maternal exposure. These results could be beneficial in understanding the long-term effects of SHS or eCig exposure in the development of placental diseases.

## 1. Introduction

The placenta is the primary site of nutrient and gas exchange between the mother and the fetus, with its proper function being important for a successful pregnancy. Environmental stresses during pregnancy have been increasingly recognized for their potential to compromise placental function, leading to conditions such as placental insufficiency, the development of intrauterine growth restriction (IUGR), and preeclampsia (PE) [1].

IUGR impacts fetal and neonatal morbidity and mortality and can lead to many other complications such as perinatal hypoxia and asphyxia, cerebral palsy, and persistent pulmonary hypertension of the newborn [2,3]. IUGR pregnancies are characterized by decreased fetal and placental weights and placental complications such as impaired trophoblast invasion, as well as increased trophoblast apoptosis [4,5]. PE is an obstetric complication characterized by high blood pressure after the 20th week of pregnancy, 140 mm Hg (systolic) or 90 mm Hg (diastolic), and production of protein in the urine (≥300 mg in 24 h) [6,7,8]. Preexisting medical conditions, such as diabetes, obesity, and hypertension, are known to be risk factors for the development of PE. 

As in IUGR, PE placentas are characterized by several pathologic findings, including decreased trophoblast invasion, increased placental trophoblast apoptosis, and increased placental inflammation [4,5,9]. Cigarette smoking during pregnancy may be the single most important avoidable cause of adverse pregnancy outcomes including increased infant mortality rates and the development of IUGR and PE [10,11,12,13]. More recently, passive secondhand smoke (SHS) exposure has been associated with adverse pregnancy consequences, including the development of IUGR, increased risk of newborn orofacial clefting, elevated risks of wheeze development in newborns, and even learning difficulties [14,15,16,17]. In addition, IUGR babies are linked to long-term sequelae of diseases, including adult hypertension, pulmonary complications, heart disease, stroke, and diabetes [17,18,19,20,21]. 

Although some information is known about SHS and its effect in placental disease, little information is known about placental complications in pregnancies exposed to electronic cigarettes (eCigs). Recently, the use of eCigs as a “healthier” alternative to traditional tobacco smoking has become more widespread. Although these methods are proposed to be healthier, recent research has shown adverse health effects associated with eCig use, including bronchitis, mouth/throat irritation, headaches, nausea, airway obstruction, bronchospasm, inflammation, and cardiovascular effects (elevated heart rate, blood pressure, and vessel stiffness). 

The objective of the current study was to determine the effects of SHS or eCig exposure in two important times of pregnancy (before the start of trophoblast invasion (6 days; starting at 12 days of gestation) and shortly after invasion has already started (4 days; started at 14.5 days of gestation)) and to determine inflammatory markers in the placenta of treated animals as compared to controls.

## 2. Methods

### 2.1. Animals and Tissue Preparation

C57 Black 6 (C57BL/6) mice were obtained from Jackson Laboratories (Bar Harbor, ME, USA). Mice were maintained on a 12-h light/dark cycle at the animal facility and supplied with food and water ad libitum. After a positive pregnancy, mice were exposed to SHS or eCigs using a nose-only *InExpose* smoking system (Scireq, Montreal, QC, Canada). Pregnant mice were placed in the *InExpose* system at two different gestational ages (starting at E12.5, or E14.5 until E18.5). For SHS, exposure consisted of a computer-controlled puff generated from 6 3R4F research cigarettes (Kentucky Tobacco Research and Development Center, University of Kentucky, Lexington, KY, USA) once each minute, resulting in 10 s of SHS followed by 50 s of fresh air during a 10-min period. For eCigs, the system generated computer-controlled puffs of eCigs (cinnamon flavor + 6 mg of nicotine) once each minute to a constant flow of 80 mL. The smoke and vapor challenge chosen in the present study was associated with a good tolerance of mice to the SHS or eCig sessions and an acceptable level of particulate density concentration according to the literature. Animals were separated into five groups (n = 6 each): wild-type and room air (Cntrl), wild-type and SHS 6 days of treatment (SHS 6 days), wild-type and SHS 4 days of treatment (SHS 4 days), wild-type and eCigs 6 days of treatment (eCig 6 days), and wild-type and eCigs 4 days of treatment (eCig 4 days). Necropsies happened at E18.5, and placentas and fetuses were weighed. Proteinuria levels were also determined at this stage of pregnancy. Placental tissues were then snap-frozen in liquid nitrogen for protein analysis.

### 2.2. Blood Pressure

Blood pressure was measured using the CODA monitoring system (CODA tail-cuff blood pressure system) from Kent Scientific (Torrington, CT, USA). This system consists of an occlusion tail cuff with a fully automated controller and heating pad. The animals were restrained by a medium-sized clear column with a moving head joint that was made by Kent Scientific Corp. Restriction was used for 5 min while taking blood pressure measurements. These measurements were performed every other day on an electric warming pad to detect if there was any effect on the blood pressure in the treated and control animals.

### 2.3. Proteinuria

Levels of proteinuria were determined using a dipstick (Siemens Health Care Diagnostics, Tarrytown, NY, USA) approach to confirm PE. Briefly, urine was collected at the time of necropsy and placed on the stick, and development of color was evaluated. Categories included negative, trace, +1 (30 mg/dL), +2 (100 mg/dL), +3 (300 mg/dL), and +4 (greater or equal to 2000 mg/dL). PE is characterized by +3 and +4 levels. The results are presented as the average number for control or treated animals showing trace, +1, +2, +3, or +4. For statical differences, treated animal average proteinuria values were compared to the proteinuria values from the controls. These results were further validated using an Albuminuria Fluorometric Assay Kit (My BioSource, San Diego, CA, USA).

### 2.4. Inflammatory Molecule Analyses

The protein concentration in each of the placenta samples was quantified using a BCA Protein Assay Kit (Thermo Fisher Scientific, Waltham, MA, USA), and 125 μg of total protein lysate per sample (control and treated for a total of n = 6 per group) was collected and divided to create two sample pools, each with a concentration of 500 μg/mL (protein samples from 3 animals were pooled for each blot; n = 3 animals per blot). Two sample pools per group were added to individual membranes from a mouse inflammation antibody array C1 and the mouse cytokine antibody array C5 (RayBiotech, Norcross, GA, USA) and allowed to incubate overnight. Biotinylated antibodies were then added to each membrane and incubated overnight, followed by a final incubation with a streptavidin-conjugated fluorescent label (Thermo Fisher Scientific, Waltham, MA, USA) to detect cytokine expression. Membranes were imaged using the Odyssey DLx Near-Infrared Fluorescence Imaging System (LI-COR, Lincoln, NE USA). Results were quantitatively analyzed using Image J (U.S. National Institutes of Health, Bethesda, MD, USA) and normalized to the array’s positive controls on each membrane. Fold inductions were calculated by comparing the ratios of the treated samples to the ratios of controls.

### 2.5. Statistical Analysis

Mean values ± S.E. per group were assessed by one- and two-way analysis of variance (ANOVA). Results with *p*-values < 0.05 were considered significant.

## 3. Results

### 3.1. Blood Pressure, Proteinuria, and Fetal and Placental Weights

**Figure 1:**
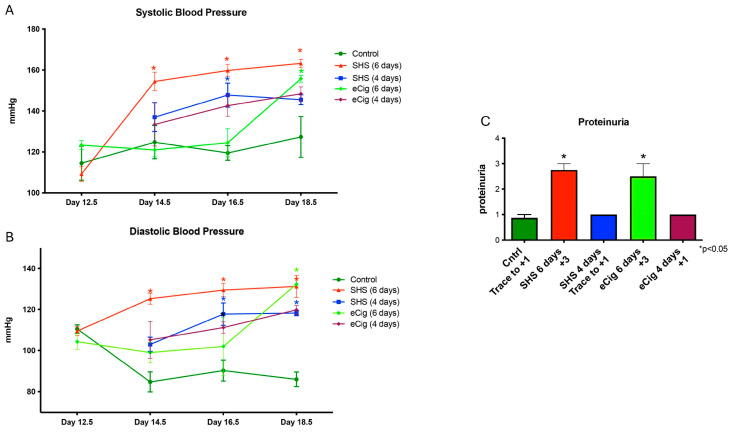
Systemic blood pressure and proteinuria during SHS or eCig treatment. There were significant increases in systolic (**A**) and diastolic (**B**) blood pressure in animals treated for six days (n = 6) with either SHS or eCigs as compared to controls. A dipstick test (n = 6) showed increased proteinuria (from +3 to +4; *p* < 0.0004) with six days of treatment with either SHS or eCigs as compared to controls (**C**). Data are shown with * *p* ≤ 0.05.

Increased blood pressure is one of the hallmarks of PE [8]. To determine if blood pressure changed with either eCig or SHS treatment, blood pressure assessments were performed. At the time of necropsy, both systolic and diastolic blood pressures were increased (*p* < 0.04) following six days of either SHS or eCig treatment (Figure 1A,B). After four days of treatment, there was only a significant increase in diastolic blood pressure with SHS treatment (Figure 1A,B). Increased proteinuria is another marker of complications with preeclampsia [8]. We observed a significant increase in proteinuria (2.5-fold increase; *p* < 0.004) associated with six days of treatment with both SHS and eCigs (Figure 1C).

**Figure 2:**
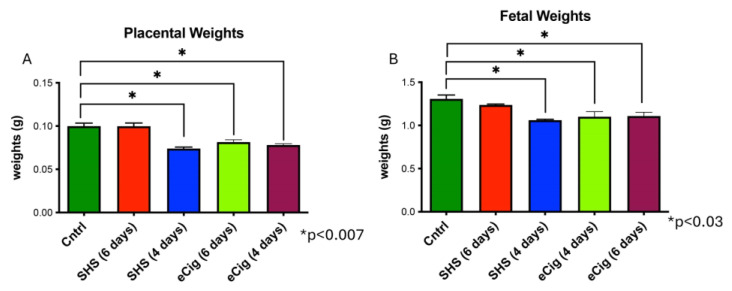
Placental and fetal weight differences during SHS and eCig exposure. A significant decrease in placental (*p* < 0.0001) and fetal weights (*p* < 0.02) was observed in four-day SHS-treated animals as compared to controls (**A**,**B**). A significant decrease in placental (*p* < 0.0008) and fetal weights (*p* < 0.0006) was observed after 6 and 4 days in the eCig-treated animals as compared to controls (**A**,**B**).

A hallmark of IUGR development is decreased placental and fetal weights [14]. Next, we determined placental and fetal weight changes during SHS and eCig treatment. During SHS treatment, placental (1.4-fold; *p* < 0.0001) and fetal weights (1.4-fold; *p* < 0.02) were decreased following four days of treatment (Figure 2A,B). In contrast, placental and fetal weights were decreased after six days (1.23-fold, *p* < 0.0008; and 1.28-fold, *p* < 0.006) and four days (1.2-fold, *p* < 0.0001; 1.2-fold, *p* < 0.005) of eCig treatment (Figure 2A,B).

### 3.2. Inflammatory Molecule Analyses

Understanding the specific inflammatory pathways and associated cytokines is crucial in characterizing both normal pregnancy and the molecular pathogenic mechanisms of conditions like preeclampsia (PE) and intrauterine growth restriction (IUGR).

#### 3.2.1. Th1, Th17, and Th2 Pathway Molecules

During pregnancy, the balance between cell-mediated immunity (Th1) and humoral immunity (Th2) is important for pregnancy outcomes. Initially, there is prevalence of Th2 cytokines during pregnancy, and this is followed by a change to Th1 predominance later in gestation [22]. Interferon gamma (INF-γ) is a key cytokine in the Th1 response, promoting cell-mediated immunity [23]. Th17 pathways are induced in parallel to Th1 and can lead to inflammation and autoimmune disease [24]. INF-γ is a proinflammatory cytokine secreted in the uterus during early pregnancy and is known to be increased in pregnancies complicated with IUGR and PE [25].

**Figure 3:**
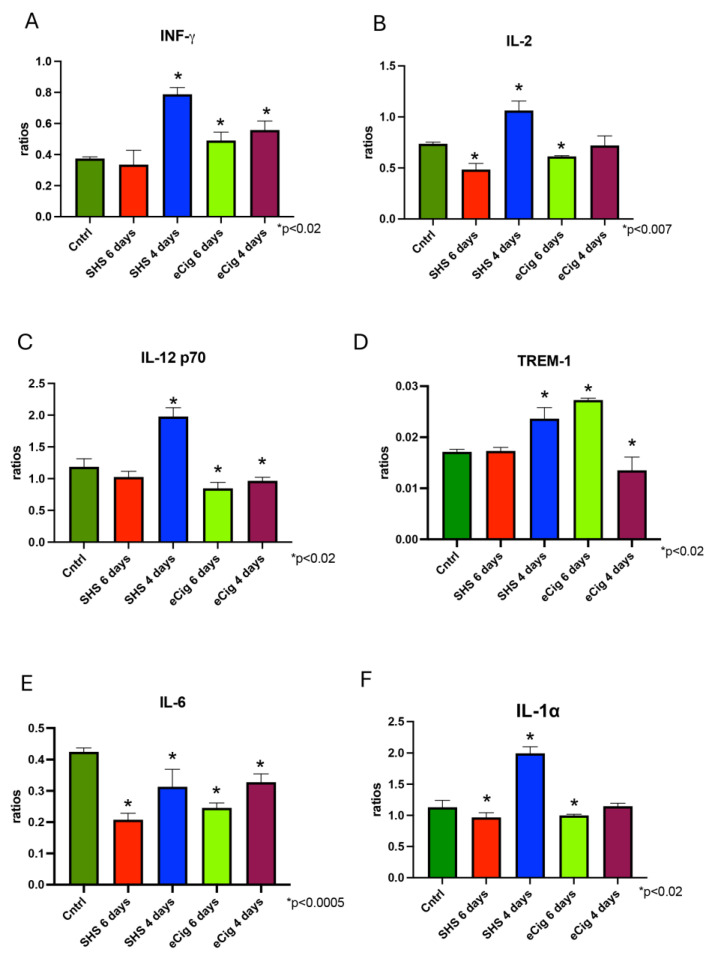
Placental Th1 and Th17 and Th2 cytokines during SHS or eCig treatment. Inflammatory mediators were determined in treated animals as compared to controls. Protein expression of INF-γ (**A**), IL-2 (**B**), IL-12 p70 (**C**), TREM-1 (**D**), IL-6 (**E**), and IL-1α (**F**) was differently regulated by length and type of treatment when compared to controls. Significant differences are noted as * *p* ≤ 0.05.

We detected increased INF-γ expression (2.1-fold; *p* < 0.0001) in the placenta after six days of SHS treatment (Figure 3A). Similarly, a placental increase was also observed at both six (1.3-fold, *p* < 0.02) and four days (1.5-fold, *p* < 0.0004) with eCig treatment (Figure 3A). IL-2 supports Th1 differentiation and proliferation. IL-2 is expressed in the syncytiotrophoblast in the human placenta, and it has been shown that in low dosages it normalizes hypertension in a mouse model of placental ischemia [26,27]. IL-2 was increased in the placenta (1.5-fold, *p* < 0.002) after four days of SHS treatment (Figure 3B). In contrast, IL-2 was decreased at four days of SHS treatment (1.4-fold, *p* < 0.002) and at six days of eCig treatment (1.2-fold, *p* < 0.02, Figure 3B). IL-12 is a major inducer of the Th1 response [28]. This cytokine has two isoforms, bioactive IL-12 p70 and regulatory IL-12 p40, that are expressed in the placenta [29,30]. Placental bioactive IL-12 p70 isoform was increased only after four days of SHS (1.7-fold, *p* < 0.002, Figure 3C). In contrast, we observed that IL-12 p70 was decreased after both six (1.4-fold, *p* < 0.009) and four days (1.2-fold, *p* < 0.02) of eCig treatment (Figure 3C). TREM-1 is a molecule that has been implicated in the propagation of the inflammatory response that is upregulated during PE [31]. Placental Trem-1 was increased following four days of SHS treatment (1.4-fold, *p* < 0.002) and with six days of eCig treatment (1.6-fold, *p* < 0.0001, Figure 3D). In contrast, we found that placental TREM-1 was decreased in the eCig animals treated for four days (1.3-fold, *p* < 0.02, Figure 3D). IL-6 cytokine is characterized by having both pro- and anti-inflammatory effects and is decreased in the PE placenta [32]. Treatment with either SHS or eCigs showed a decrease in placental IL-6 at all gestational points studied (~2.0-fold, *p* < 0.002 for six days of treatment and ~1.3-fold for four days of treatment; Figure 3E). IL-1α is a potent proinflammatory cytokine that is produced by the placenta [33]. Treatment with SHS for six days decreased IL-1α levels (1.2-fold, *p* < 0.02), while there was a 1.8-fold increase (*p* < 0.0002) in IL-1α after four days of treatment when compared to controls (Figure 3F). We found that eCig treatment decreased IL-1α (1.1-fold, *p* < 0.04) when animals were treated for six hours.

**Figure 4:**
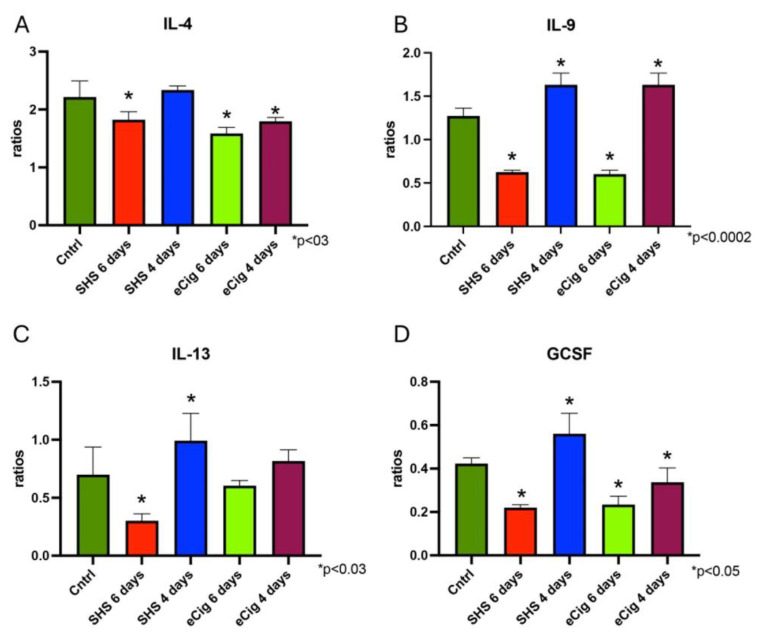
Placental Th2 cytokines during SHS or eCig treatment. Inflammatory mediators were determined in treated animals as compared to controls. Protein expression of IL-4 (**A**), IL-9 (**B**), IL-13 (**C**), and GCSF (**D**) was differently regulated by length and type of treatment when compared to controls. Significant differences are noted as * *p* ≤ 0.05.

While the Th1 path is most associated with generating an inflammatory response, Th2 is generally associated with producing an anti-inflammatory response [34]. During pregnancy, the Th2 response is dominant in order to aid fetal health and appropriate placental development [35]. IL-4 is a cytokine that functions as a potent regulator of immunity secreted by Th2 cells [36]. In the placenta, IL-4 is produced by the trophoblast cells and has been shown to be decreased during PE [37,38]. Treatment with SHS for six days showed a significant decrease in placental IL-4 (1.2-fold, *p* < 0.003), with no significant differences observed after four days of treatment as compared to controls (Figure 4A). This cytokine was also decreased in the placenta after six (1.4-fold, *p* < 0.0002) and four days (1.2-fold, *p* < 0.002) of eCig treatment as compared to controls (Figure 4A). IL-9 is a cytokine released by the trophoblast cells that is decreased in PE [39]. We observed a decrease in IL-9 levels after exposure to either SHS (2.0-fold, *p* < 0.0002) or eCigs (2.1-fold, *p* < 0.0002) in the animals treated for six days (Figure 4B). In contrast, both SHS (1.3-fold, *p* < 0.0002) and eCig (1.3-fold, *p* < 0.0002) exposure increased IL-9 in the animals treated for four days as compared to controls (Figure 4B). IL-13 closely works with IL-4, sharing many functions, including the modulation of inflammatory responses. During pregnancy, IL-13 is produced in the placenta throughout gestation, and it is increased during IUGR [40,41]. SHS-treated animals showed decreased placental IL-13 levels (2.3-fold, *p* < 0.003) after six days of treatment (Figure 4C). In contrast, a significant increase in placental IL-13 (1.4-fold; *p* < 0.03) was observed when animals were treated with SHS for four days (Figure 4C). GCSF activities include neuroprotection, cardiac cell generation and repair, and immunomodulation [42]. We observed that four days of SHS treatment increased GCSF protein levels (1.3-fold, *p* < 0.004) in the placenta of treated animals as compared to controls (Figure 4D), while animals treated with eCigs showed a decrease in placental GCSF at all treatment points (six days, 1.8-fold, *p* < 0.0004, and four days, 1.3-fold, *p* < 0.05; Figure 4D).

#### 3.2.2. Chemokine Signaling Pathway

**Figure 5:**
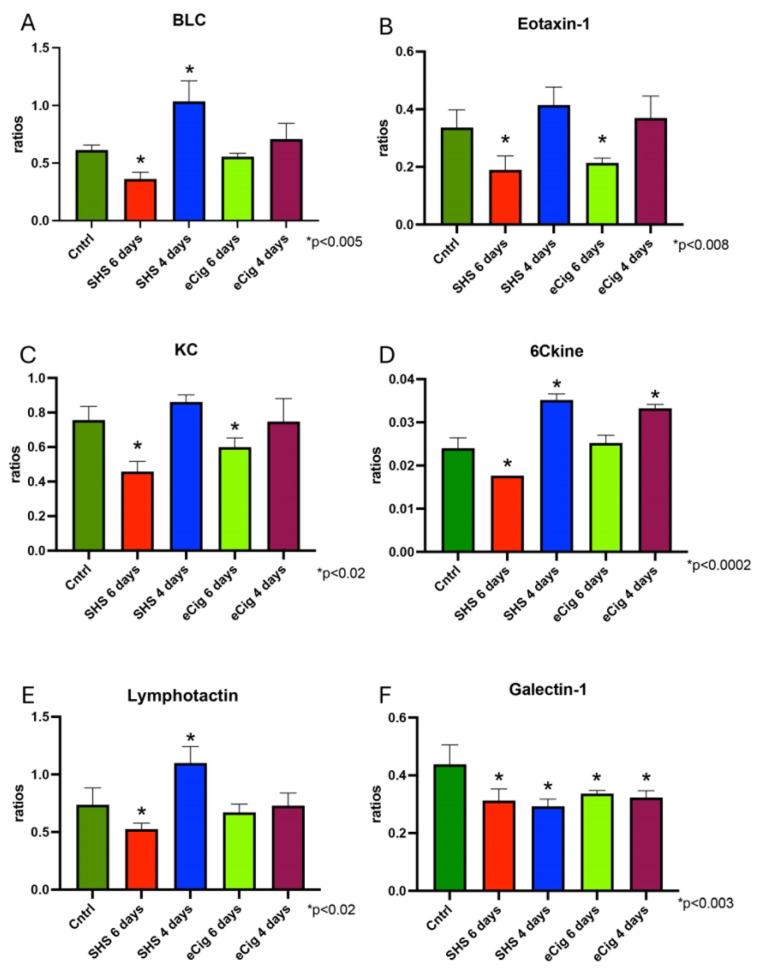
Placental chemokine signaling pathway molecules during SHS or eCig treatment. Inflammatory mediators were determined in treated animals as compared to controls. Protein expression of BLC (**A**), eotaxin-1 (**B**), KC (**C**), 6Ckine (**D**), lymphotactin (**E**), and galectin (**F**) was differently regulated by length and type of treatment when compared to controls. Significant differences are noted as * *p* ≤ 0.05.

Chemokines are secreted molecules that can act as chemoattractants, promoting the migration of nearby responding cells [43]. Inflammatory diseases, such as PE, are associated with the aberrant production of cytokines, emphasizing the role of these molecules during inflammation [43]. One of these cytokines, BLC (or CXCL13), is expressed in the placenta, and the role of this molecule during pregnancy infections has been established [44]. Interestingly, in our studies we observed a significant decrease in BCL (1.7-fold, *p* < 0.005) with six days of SHS treatment, while there was a significant increase of placenta BLC (1.7-fold, *p* < 0.0002) after four days of SHS treatment as compared to controls (Figure 5A). Eotaxin-1 is a cytokine involved in the regulation of the invasive properties of trophoblast cells [45]. We detected a decrease in placental eotaxin-1 when animals were treated for six days with either SHS (1.8-fold, *p* < 0.003) or eCigs (1.6-fold, *p* < 0.008, Figure 5B). KC (keratinocyte-derived chemokine; also known as chemokine (C-X-C motif) ligand 1 or CXCL1) is a chemokine known to aid the process of trophoblast invasion [46]. Similar to our findings on eotaxin-1 production, we observed KC production to be decreased when the animals were treated with SHS (1.7-fold, *p* < 0.0002) or eCigs (1.3-fold, *p* < 0.02) for six days (Figure 5C). 6Ckine (also known as chemokine (C-C motif) ligand 21 or CCL21) is a chemokine secreted by trophoblast cells [47]. We found that exposure to SHS decreased placental 6Ckine levels (1.5-fold, *p* < 0.0002) after six days of exposure, while we observed a significant increase (1.5-fold, *p* < 0.0002) following four days of exposure (Figure 5D). Lymphotactin (XCL10) is found in the placenta and has been shown to be involved in trophoblast invasion [48]. This chemokine was only affected by SHS treatment, which showed a placental lymphotactin decrease (1.4-fold, *p* < 0.02) after six days of treatment, while we observed an increase (1.5-fold, *p* < 0.0004) after four days of exposure (Figure 5D). Galectin-1 is involved in modulating immune responses. It is expressed in trophoblast cells and it is increased during normal pregnancy [49]. Both SHS and eCig treatments decreased placental levels of Galentin-1 at both four and six days of treatment (~1.4-fold, *p* < 0.003, Figure 5E).

#### 3.2.3. TNF Family Signaling Pathway

**Figure 6:**
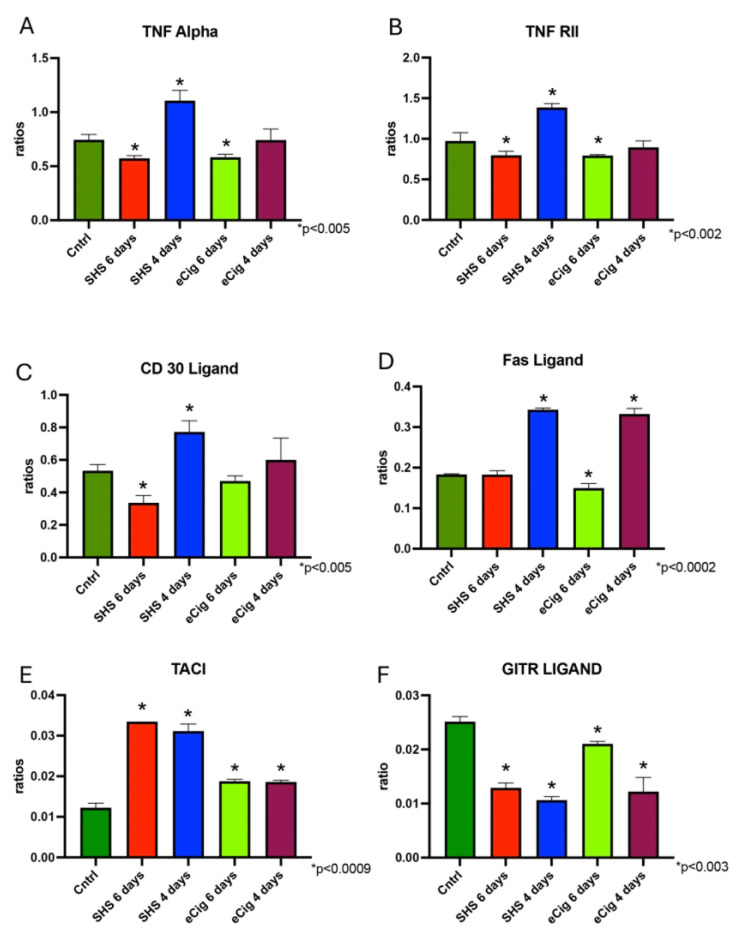
Placental TNF family signaling pathway molecules during SHS or eCig treatment. Inflammatory mediators were determined in treated animals as compared to controls. Protein expression of TNF-α (**A**), TNF RII (**B**), CD 30 ligand (**C**), Fas ligand (**D**), TACI (**E**), and GITR ligand (**F**) was differently regulated by length and type of treatment when compared to controls. Significant differences are noted as * *p* ≤ 0.05.

The TNF family controls numerous immune functions and other processes, including embryonic development processes and even cancer [50]. TNF-α is a major regulator of inflammatory responses in which the pathway’s activation can lead to various cellular responses, including cell survival, differentiation, and proliferation [51,52,53]. TNF-α was increased in the four-day SHS-treated animals (1.5-fold; *p* < 0.0002), while a decrease in this molecule was seen when the animals were treated for six days with either SHS (1.3-fold; *p* < 0.004) or eCigs (1.3-fold; *p* < 0.005) as compared to controls (Figure 6A)**.** This same pattern was observed with TNR II in these experiments (Figure 6B). CD30 ligand is a member of the TNF family and plays a role in immune responses. We found that CD30L was decreased following six days of SHS exposure (1.6-fold, *p* < 0.005), while we observed an increase after four days of SHS treatment (1.4-fold, *p* < 0.0006) in the placenta of treated animals as compared to controls (Figure 6C). Fas ligand (FasL) binds to its receptor Fas, leading to the induction of apoptosis [54]. This pathway is crucial for the regulation of the immune system, including the elimination of inflammatory cells to resolve inflammation [54]. We observed increased FasL levels after four days of exposure to either SHS (1.9-fold, *p* < 0.0002) or eCigs (1.9-fold, *p* < 0.0002) in the placenta of treated animals as compared to controls (Figure 6D). In contrast, we found that the level of placental FasL was decreased only after six days of eCig treatment (1.2-fold, *p* < 0.0002; Figure 6D). TACI is a regulator of inflammation present in the term placenta [55]. We found that TACI protein levels were increased after either four or six days of SHS (2.7-fold and 2.5-fold, *p* < 0.0.0002) or eCig (both at 1.5-fold, *p* < 0.0002) treatment as compared to controls (Figure 6E). Glucocorticoid-induced TNF-related ligand (GITRL) is a member of the TNF superfamily that plays a role in immune cell signaling, activation, and survival [56]. We found that exposure to either SHS or eCigs decreased placental GITRL at all time points studied (between 1.2- and 2.0-fold, *p* < 0.003, Figure 6F).

#### 3.2.4. Other Associated Proteins

**Figure 7:**
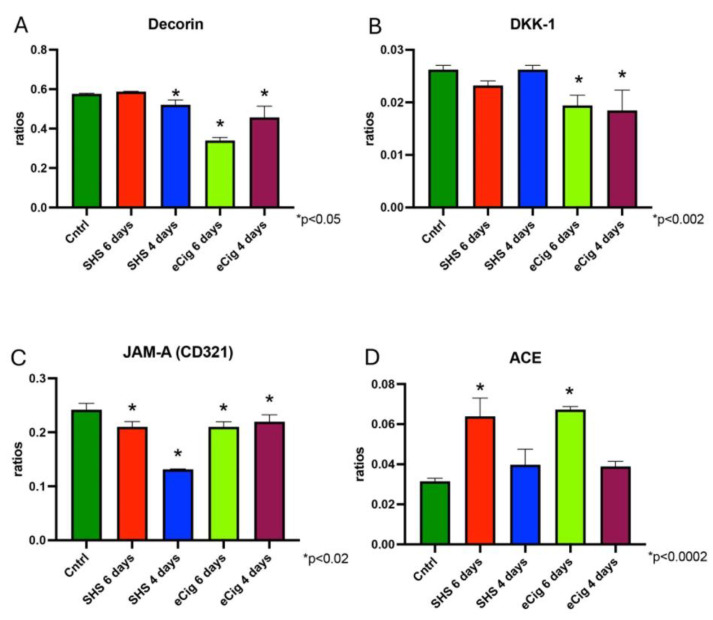
Other placenta-related inflammatory molecules during SHS or eCig treatment. Inflammatory mediators were determined in treated animals as compared to controls. Protein expression of decorin (**A**), DKK-1 (**B**), JAM-A (**C**), and ACE (**D**) was differently regulated by length and type of treatment when compared to controls. Significant differences are noted as * *p* ≤ 0.05.

Decorin is an extracellular matrix protein and has been implicated in inflammatory responses, autophagy, angiogenesis, cell cycles, wound healing, and fibrosis [57]. We found that decorin levels did not change when animals were exposed to SHS for six days; in contrast, this protein showed a modest decrease in production (1.1-fold, *p* < 0.05) when animals were exposed to SHS after trophoblast invasion started (four days of treatment, Figure 7A). We also observed that eCigs decreased placental decorin when animals were exposed for either six (1.7-fold, *p* < 0.0002) or four days (1.3-fold, *p* < 0.0002, Figure 7A). DKK-1 is a known inhibitor of the Wnt signaling pathway, which is involved in a wide range of processes and has been linked to immunosuppressive effects and fibrosis [58], and has been shown to be decreased during fetal growth restriction [59]. We found that placental DKK-1 levels were decreased after eCig exposure after both four (1.7-fold, *p* < 0.002) and six days (1.3-fold, *p* < 0.0004) of exposure, while no changes were detected when animals were treated with SHS (Figure 7B). JAM-A (CD321) is involved in cell adhesion and tight junction formation. It can influence inflammatory responses by regulating leukocyte migration and barrier function [60]. Similarly to E-Cadherin, we found that placental JAMA-A was decreased at all study periods when animals were treated with SHS (1.1-fold, *p* < 0.002; 1.8-fold, *p* < 0.0002) or eCigs (both at 1.1-fold, *p* < 0.02, Figure 7C). ACE is mainly known for its role in blood pressure regulation through the renin–angiotensin system. It converts angiotensin I to angiotensin II, a potent vasoconstrictor, but also has roles in inflammation and fibrosis [61]. Our results showed that exposure to SHS decreased placental ACE at both of the studied time points (1.4-fold, *p* < 0.0005; 1.9-fold, *p* < 0.0002, Figure 7D). We also showed that ACE was also decreased in the animals treated with eCigs at all time points studied (1.3-fold, *p* < 0.004; 2.0-fold, *p* < 0.0002, Figure 7D).

## 4. Discussion

Environmental stresses during pregnancy have significant implications for maternal and fetal health. Understanding the impact of environmental stresses on pregnancy is crucial, given their potential to contribute to conditions such as intrauterine growth restriction (IUGR) and preeclampsia (PE), which are critical determinants of neonatal and long-term health. Placental insufficiency arises from a multitude of factors, including abnormal placental development, insufficient uteroplacental blood flow, and impaired placental nutrient and gas exchange. A hallmark of these conditions is defective trophoblast invasion and spiral artery remodeling, which are essential for establishing adequate maternal blood flow to the placenta [62]. This failure in vascular remodeling leads to chronic hypoxia and oxidative stress within the placenta, altering its structure and function [63]. In our study, we wanted to determine the effects of secondhand smoke (SHS) and e-cigarette treatment (eCig) at two specific points, before the start of trophoblast invasion (starting at 12.5 days of gestation; six days of exposure) and after this invasion started (starting at 14.5 days of gestation; four days of exposure). Previous studies in our laboratory showed the development of IUGR when animals were treated for four days (14.5—18.5 days of gestation) with SHS [14]. In our experiments, we first wanted to determine if SHS treatment affected the blood pressure of pregnant mice at the studied gestational points. Maternal blood pressure was increased in the animals treated with SHS before the start of trophoblast invasion (six days of treatment). In contrast, four days of SHS treatment increased only diastolic blood pressure but not systolic in the treated mothers as compared to controls. Seeing increased diastolic pressure with IUGR during four days of SHS treatment was surprising, but reports have shown that IUGR can also be characterized by increased blood pressure [64]. Besides blood pressure, an increase in proteinuria is another characteristic of PE pregnancies. Proteinuria increased after six days of treatment with both SHS and eCigs. This suggests the presence of at least some PE characteristics at these gestational points. Previously, we showed decreased fetal and placental weights with SHS treatment, leading to IUGR development when animals were treated for four days. We next determined placental and fetal weights with the different treatments. As expected, we observed decreased placental and fetal weights after four days of SHS treatment. Interestingly, a decrease in placental and fetal weights was also observed when animals were treated with eCigs at both four and six days of treatment. These results suggest that animals treated with SHS before the start of trophoblast invasion show characteristics of the development of PE, while six days of exposure to eCigs resulted in PE with the IUGR phenotype. Furthermore, these results suggest that impaired trophoblast invasion could be involved in the decreased placental and fetal weights observed during development of these obstetrics complications and that these weights could also have been affected by the length and the different type of treatments observed for these animals. As observed previously, treatment for four days after the start of trophoblast invasion induced the IUGR phenotype when SHS was administered. A similar outcome was also observed in the animals exposed to eCigs for four days. Although a link between cigarette smoke and obstetric complications has been established, not much information is available about the effect of eCigs during pregnancy. To our knowledge, this is the first report of the in vivo effects of electronic cigarette use during pregnancy. Furthermore, we studied the in vivo effects of both eCigs and SHS at different stages of pregnancy. This suggests a role of the gestational period in the development of obstetric complications associated with exposure to these contaminants.

PE and IUGR are obstetric complications associated with increased inflammation [65,66]. We next decided to study inflammatory molecules associated with the development of obstetric disease with SHS and eCig treatment.

### 4.1. Th1, Th17, and Th2 Pathways and Proteins

Normal pregnancies are associated with a Th2 immune response, while a Th1 response is considered dangerous for pregnancy. In fact, the development of preeclampsia has been implicated with an increase in the Th1 response [67]. Interferon gamma (INF-γ) is a proinflammatory cytokine involved in Th1 responses. Pregnancies associated with PE have been characterized by increased INF-γ, and the role of regulation by this molecule on trophoblast invasion has been shown [68,69]. We observed increased INF-γ coinciding with the PE-like symptoms observed during the 6 days of treatment with eCigs. This suggests that, like in human PE, this cytokine could be involved in the development of this obstetric complication. Interestingly, we observed decreased INF-γ after six days of exposure to SHS. This was a surprising result, but studies on other body systems have shown that INF-γ responses are decreased in smoking environments. This suggests that the decrease observed in our system could be a response to the treatment used. In the animals experiencing only IUGR symptoms (after four days of exposure), INF-γ was not significantly affected by SHS, while it was increased by eCigs. This is interesting, as a previous report had established no changes to INF-γ during IUGR [41]. However, we did see an increase with eCigs, suggesting that an immune response dependent on treatment leads to the development of this obstetric complication.

IL-2 is a Th1 cytokine involved in the activation of T cells. Interestingly, studies suggest that increased IL-2 is associated with decreased blood pressure in a model of reduced uterine perfusion pressure [70]. Both treatments (SHS and eCigs) induced a significant decrease in IL-2 after six days of treatment. This correlates with the increased blood pressure present at this time point, and suggests the possible role of IL-2 downregulation in animals that are experiencing PE-like symptoms. Treatment with SHS for four days increased placental IL-2 levels, while no differences were observed with eCig treatment in these animals. IL-12 p70 is characterized by the production IFN-γ. It is also implicated in the pathogenesis of autoimmune diseases where Th1 responses are dysregulated [71]. During SHS treatments (four and six days), IL-12 p70 follows the same pattern as IFN-γ with the same treatment. This suggests that IL-12 p70 may be involved in the regulation of IFN-γ during exposure to smoke-related environments. Both eCig treatments (four and six days) decreased placental IL-12 p70. This was unexpected, as IFN-γ levels were increased in the same periods studied. This suggests that perhaps another mechanism, independent of IL-12 p70, could be regulating IFN-γ during this treatment. Although we observed a similar decrease in some of the Th2 cytokines studied, TREM-1 and IL-1-α were affected differently. TREM-1 is a cell surface molecule involved in the promotion of inflammation by enhancing the production of proinflammatory cytokines [72,73]. Previous reports have shown increased TREM-1 in the preeclamptic placenta, leading to increased trophoblast inflammation [31,74]. We observed increased TREM-1 in the placenta of animals treated with eCigs for six days. This supports, as in humans, a possible role of TREM-1 during PE.

There were no detected changes in TREM-1 in the animals treated with SHS for the same gestational time. This suggests that perhaps TREM-1 expression could be influenced differently by these treatments. A possible explanation could be that TREM-1 is expressed in severe PE, and perhaps the PE observed in these treatments could be different. IL-1α is present in most tissues and cells and can be released upon cell injury, initiating the inflammatory response [75,76]. We only observed increased IL-1α after four days of SHS treatment. Previous human studies showed increased plasma levels of IL-1α during IUGR [77]. Our results suggest that perhaps the increase in placental IL-1α could be associated with the increased plasma levels observed during IUGR.

For the animals experiencing PE-like symptoms, we observed a general decrease in the Th2-associated cytokines studied (IL-4, IL-9, IL-13, GCSF). This supports the idea of a decreased Th2 response during the development of PE. In contrast, SHS-induced IUGR pregnancies had a general increase in Th2 cytokines, which could be a response to the stress found in the placentas or fetuses at these gestational points.

### 4.2. Chemokine Signaling Pathway

Chemokines are small-molecule cytokines expressed in the trophoblast cells participating in trophoblast invasion, decidualization, and immune cell recruitment [78]. Chemokines and galectins play pivotal roles in immune regulation and tissue remodeling during pregnancy. BLC (or CXCL13) is a cytokine involved in inflammatory responses. In our studies, this protein was only affected by SHS; this supports reports that identify BLC as a cytokine involved in lung cancer within a smoking environment. This is interesting as BLC is usually recognized during infection, but we observed placental changes in our experiments. We only detected increased BLC when animals were treated with SHS after the start of trophoblast invasion. This is important because not much information is present about this cytokine in the placenta during IUGR, suggesting a possible novel role of this cytokine during the placental inflammation observed during this disease. Interestingly, a decrease in this protein was detected in the animals with PE-like symptoms (six days of SHS). Although studies showed the role of this molecule in implantation and trophoblast migration, our studies also suggest a role for BLC in the development of this placental disease.

Eotaxin-1 is a potent chemoattractant for eosinophils and is involved in the processes of angiogenesis [78]. In our studies, both molecules were affected after six days of treatment with both SHS and eCigs. In both cases, they were significantly decreased when PE-like symptoms were present. In the case of eotaxin-1, studies have shown a correlation between this molecule and increased trophoblast invasion, and its levels are decreased during PE [45,79]. The decrease observed in our studies supports previous findings related to the presentation of PE-like symptoms in these animals. Similarly, KC was decreased in these animals. Although a direct correlation between KC and PE has not been established, it is known that this disease is associated with decreased angiogenesis and decreased angiogenic growth factor, suggesting that perhaps a KC decrease could be a factor contributing to the decreased angiogenesis during PE. The 6Ckine cytokine regulates the invasion of trophoblast cells, and is known to be decreased during PE [47]. We observed a decrease in this cytokine only in SHS-induced PE (six days of treatment) during treatment with SHS, but not during eCig treatment. In contrast, 6Ckine was decreased with both treatments in the IUGR animals. From our studies, we can conclude that perhaps 6Ckine is not involved during PE when IUGR is also present.

Lymphotactin is an inflammatory cytokine that also has a role in increasing trophoblast invasion [48,80]. It is increased during smoke-induced inflammation in the lungs [81]. In our experiments, SHS affected lymphotactin differently on the two gestational treatment days. While lymphotactin decreased after six days of SHS treatment, it was significantly increased after four days of SHS treatment. Perhaps the timing of treatment affected the role of lymphotactin in our experiments. Furthermore, we can hypothesize that starting the SHS treatment prior to trophoblast invasion led to a decrease in this cytokine, which may be contributing to the decreased trophoblast invasion that characterizes PE. In contrast, when treatment began after the start of trophoblast invasion, this cytokine could be involved in the regulation of inflammation at this gestational point. Galectin-1 regulates processes including the maternal immune response, regulation of trophoblast invasion, and migration [82]. We detected decreased galectin-1 levels at all time points studied and with either treatment, which supports the previously reported decrease during IUGR and PE [82,83].

### 4.3. TNF Family Signaling Pathway

TNF-α is a major regulator of the inflammatory response in many cells. In pregnancy complicated by PE or IUGR, maternal serum levels of TNF-α are increased as compared to controls [30,84]. In our experiments, we tested TNF-α and TNF RII protein levels in the placenta of treated animals as compared to controls. Both proteins showed the same pattern of increased levels in SHS-induced IUGR, while they were decreased in the placenta of the PE animals. Interestingly, reports on human PE placentas showed that there were no significant differences in placental TNF-α protein during PE. Our results differ from those observed during human PE, which could have resulted from the PE induced in these animals being caused by these exposures, which possibly affect TNF-α levels differently. Previously, our laboratory showed increased placental TNF-α during SHS-induced IUGR [14]. Our experiment confirmed these results and extended the discovery of increased TNF-α in eCig-induced IUGR. TNFR II is predominantly found in immune cells, and the fact that its expression mimics that of TNF-α suggests the possible role of these molecules in the inflammatory response in the treated animals as compared to controls.

CD30L is expressed in the early and late placenta [85]. Serum levels of this molecule are decreased during PE, but only minimal information is known about this molecule during IUGR. We found that CD30L was affected only by SHS treatment. Following this treatment, we saw decreased CD30L levels during PE, but increased levels during IUGR in the placenta of treated animals as compared to controls. It is known that CD30L has increased levels in other organs within smoking environments. Perhaps gestational age and treatment time influenced the different expression of CD30L in the placenta of the SHS-treated animals. Fas ligand (FasL) initiates apoptosis by binding to its surface receptor Fas. Past studies have shown decreased FasL levels during PE-IUGR and increased FasL levels during IUGR in humans [86,87]. Our results support the prior findings, since we observed increased FasL in the IUR-PE animals and increased FasL during IUGR. These results demonstrate the similarity of our models to human diseases.

TACI is a molecule involved in B-cell activation and plasma cell survival. Placental TACI may play a role in modulating immune responses during pregnancy complications such as preeclampsia and IUGR [55]. Although not much information is present about the role of this molecule in the placenta, we detected increased TACI at all of the time points studied. This suggests a role for the protein during the inflammation present in the placenta during the development of obstetric complications such as PE and IUGR. GITR/GITRL is a critical modulator of T-cell function and has been shown to influence placental immune tolerance. Alterations in GITRL expression are associated with immune maladaptations [88]. We observed decreased GITRL at all the time points in this study. This suggests that perhaps these maladaptations could potentially contribute to the inflammatory responses characterized in these conditions.

### 4.4. Other Associated Proteins

PE and IUGR often have overlapping pathophysiological features, including abnormal placental development and function, where immune responses play a critical role. Molecules such as decorin, Dickkopf-1 (DKK-1), and Junctional Adhesion Molecule A (JAM-A) are implicated in the modulation of these immune responses. Decorin is involved in cellular signaling, with differential expression in normal and complicated pregnancies. During PE, decorin has been reported to be increased in the decidual cells, but this was not observed in the placental trophoblast [89,90]. We did not see any differences in the PE animals treated with SHS. In contrast, we observed a decrease in this molecule in the PE animals treated with eCigs. Although we observed similar results to those in human PE with SHS, the decrease observed with eCigs suggests a treatment-dependent expression of this molecule in our two different models of PE. Placental decorin is decreased during fetal growth restriction. This same patten was observed in our IUGR mice, validating the similarity of our results with human IUGR disease. DKK-1, an inhibitor of the Wnt/β-catenin signaling pathway, plays a critical role in cell differentiation and proliferation. In these experiments, levels of DKK-1 were affected in the eCig-treated animals. With this treatment, we observed decreased levels of this protein at four and six days of treatment. Previous reports showed decreased serum levels of DKK-1 in IUGR pregnancies, and it is associated with increased invasion of trophoblast cells [59,91]. Our results are interesting as we did observe decreases in our animals treated with eCigs, but no differences were present in SHS-treated animals. This suggests that perhaps a role for this protein is present during pregnancy diseases and that it could be related to the characteristic trophoblast invasion decrease observed during this pregnancy complication. The differences observed with the treatments could be attributed to different pathways leading to these diseases depending on the treatment used.

JAM-A is involved in tight junction assembly and immune response regulation. While specific research on JAM-A’s role in preeclampsia and IUGR is very limited, we observed decreased JAM-A levels in the placenta of the animals treated with either SHS or eCigs at all gestational times studied. Our results suggest the potential impact of this protein on placental immunology and pathology that will need be addressed in future studies. ACE is crucial for the conversion of angiotensin I to angiotensin II, a peptide that regulates blood pressure and fluid balance. Decreasing ACE can be involved in the reduction of hypertension [92]. ACE was increased in both SHS- and eCig-induced PE in our studies. This suggests the role of ACE in the increased blood pressure observed in these animals.

We conclude that detrimental effects of SHS or eCigs coincide with the length of maternal exposure. We confirmed that four days of SHS exposure resulted in metrics common to IUGR, while six days of SHS exposure more closely resembled PE pathology. In terms of eCigs, six days of exposure resembles both PE and IUGR pathology, while four days of eCig treatment resembles IUGR pathology. Furthermore, we identified immune-related proteins that could play a role in the increased inflammation observed during these obstetric complications. These results could be beneficial in helping us understand the long-term effects of SHS or eCig exposure and the development of placental diseases, providing insights into placental disease progression, and clarifying possible avenues for alleviating placental complications during these exposures.

## Data Availability

The original contributions presented in the study are included in the article, further inquiries can be directed to the corresponding author/s.
